# Deoxyribonucleic Acid 5-Hydroxymethylation in Cell-Free Deoxyribonucleic Acid, a Novel Cancer Biomarker in the Era of Precision Medicine

**DOI:** 10.3389/fcell.2021.744990

**Published:** 2021-12-10

**Authors:** Ling Xu, Yixin Zhou, Lijie Chen, Abdul Saad Bissessur, Jida Chen, Misha Mao, Siwei Ju, Lini Chen, Cong Chen, Zhaoqin Li, Xun Zhang, Fei Chen, Feilin Cao, Linbo Wang, Qinchuan Wang

**Affiliations:** ^1^ Department of Surgical Oncology, Sir Run Run Shaw Hospital, Zhejiang University, Hangzhou, China; ^2^ School of Medicine, Zhejiang University, Hangzhou, China; ^3^ Department of Thyroid and Breast Surgery, Taizhou Hospital of Zhejiang Province Affiliated to Wenzhou Medical University, Luqiao, China

**Keywords:** 5-hydroxymethylcytosine, cell-free DNA, ten-eleven translocase, liquid biopsy, cancer biomarker

## Abstract

Aberrant methylation has been regarded as a hallmark of cancer. 5-hydroxymethylcytosine (5hmC) is recently identified as the ten-eleven translocase (ten-eleven translocase)-mediated oxidized form of 5-methylcytosine, which plays a substantial role in DNA demethylation. Cell-free DNA has been introduced as a promising tool in the liquid biopsy of cancer. There are increasing evidence indicating that 5hmC in cell-free DNA play an active role during carcinogenesis. However, it remains unclear whether 5hmC could surpass classical markers in cancer detection, treatment, and prognosis. Here, we systematically reviewed the recent advances in the clinic and basic research of DNA 5-hydroxymethylation in cancer, especially in cell-free DNA. We further discuss the mechanisms underlying aberrant 5hmC patterns and carcinogenesis. Synergistically, 5-hydroxymethylation may act as a promising biomarker, unleashing great potential in early cancer detection, prognosis, and therapeutic strategies in precision oncology.

## Introduction

Carcinogenesis is a multistep and complex process in which both genetic and epigenetic aberrations play substantial roles (Feinberg et al., 2016; Thomson and Meehan, 2017). 5-methylcytosine (5mC) is a major form of DNA modification observed in most mammals’ CpG (Cytosine-phosphate-Guanine) dinucleotides (60–80%) (Smith and Meissner, 2013). The alteration of 5mC in DNA methylation patterns has been considered as a hallmark of cancer. Global hypomethylation leads to genomic instability, whereas hypermethylation at promoter regions leads to transcriptional silencing of tumor suppressor genes (TSGs), both of the above contributing significantly to the carcinogenesis (Sandoval and Esteller, 2012; Berdasco and Esteller, 2019). Additionally, hypomethylation and hypermethylation can regulate target gene expression by activating or silencing enhancers, respectively (Clermont et al., 2016; Ehrlich, 2019). These processes are dysregulated in many human cancers, leading to cellular de-differentiation and malignant transformation (Taberlay et al., 2014; Clermont et al., 2016). Along with 5mC, 5-hydroxymethylcytosine (5hmC) has been recently recognized as a stable modification that has multiple roles from cell pluripotency to carcinogenesis rather than merely intermedium of DNA demethylation (Putiri et al., 2014; Moen et al., 2015; López et al., 2017).

**FIGURE 1:**
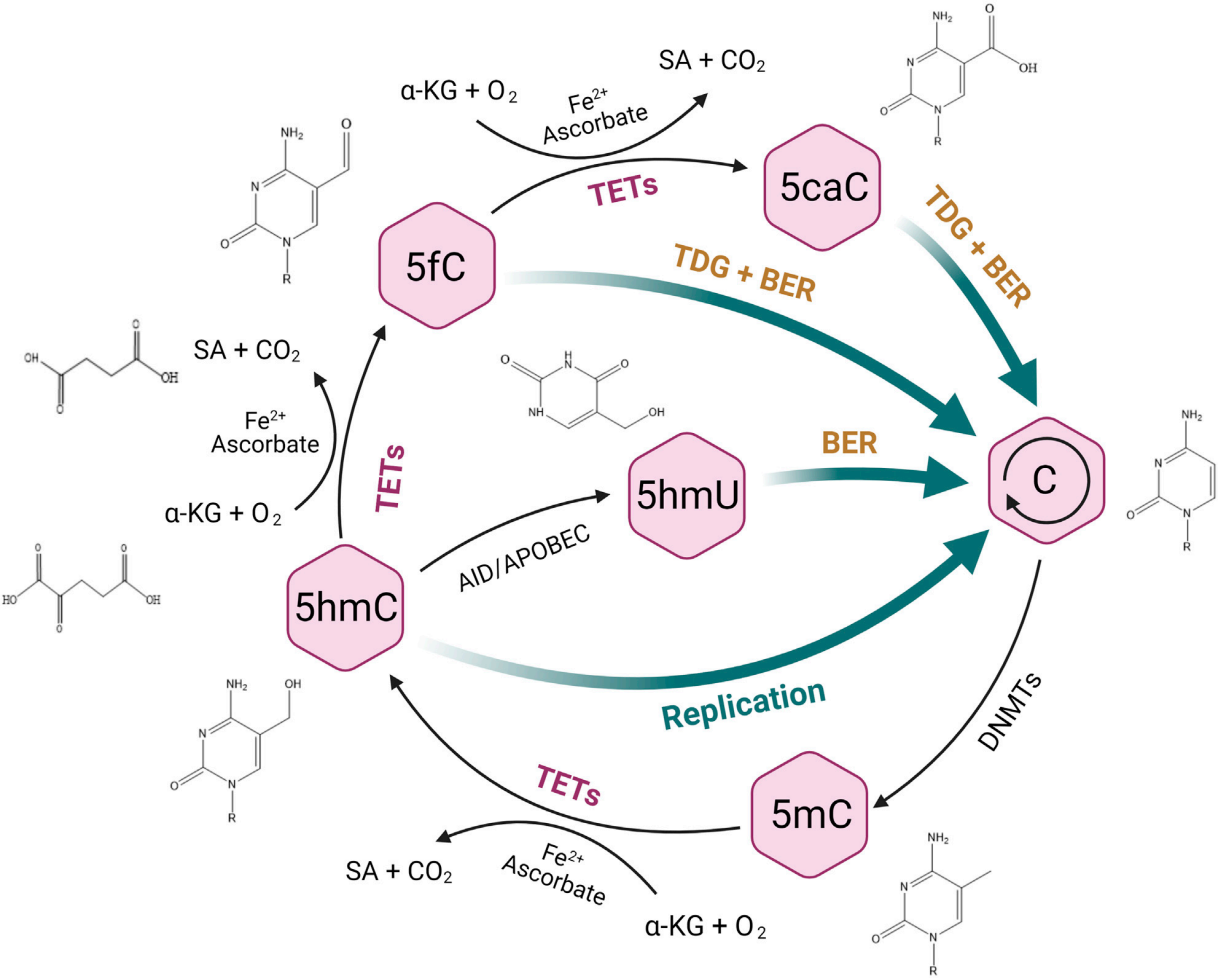
TET-mediated DNA demethylation process. TET enzymes can oxidize 5-methylcytosine (5mC) to 5-hydroxymethylcytosine (5hmC), and further to 5-formylcytosine (5fC) and 5-carboxylcytosine (5caC), followed by the excision of 5fC and 5caC via thymine DNA glycosylase (TDG) and the replacement of unmethylated cytosine through base-excision repair (BER). 5hmC can also be deaminated to 5-hydroxymethyluracil (5hmU) by AID/APOBEC enzymes and subsequently to an unnmethylated status by BER.

5hmC is the most abundant oxidation product of 5mC during active DNA demethylation that is mediated by ten-eleven translocase (TET) (Kriaucionis and Heintz, 2009; Tahiliani et al., 2009). TET enzymes oxidize 5mC to 5hmC, and further to 5-formylcytosine (5fC) and 5-carboxylcytosine (5caC) (Ito et al., 2011; He et al., 2011) in a 2-oxoglutarate (2-OG) and Fe (II)-dependent manner (Kohli and Zhang, 2013; Koivunen and Laukka, 2018) (Figure 1). 5hmC is found enriched over gene bodies, promoters, and enhancers upstream of transcriptional start sites, suggesting that 5hmC plays a role in the regulation of gene expression (Thomson and Meehan, 2017). 5hmC displays a cell- or tissue-specific distribution in different malignancies, and reduced global 5hmC levels were identified in multiple cancers (Lian et al., 2012; Orr et al., 2012; Chen et al., 2016; Tong et al., 2019; Wang et al., 2020), implying that 5hmC may have a promising value in cancer screen, diagnosis, progression and survival (Lian et al., 2012; Zhang et al., 2016a; Zhong et al., 2018). Moreover, 5hmC is suggested as a potential target to synergize with conventional treatment of cancer (Thomson and Meehan, 2017). Thus, 5hmC could be considered as a robust biomarker in clinical practice and promote precision medicine, although awaiting further validation.

**FIGURE 2:**
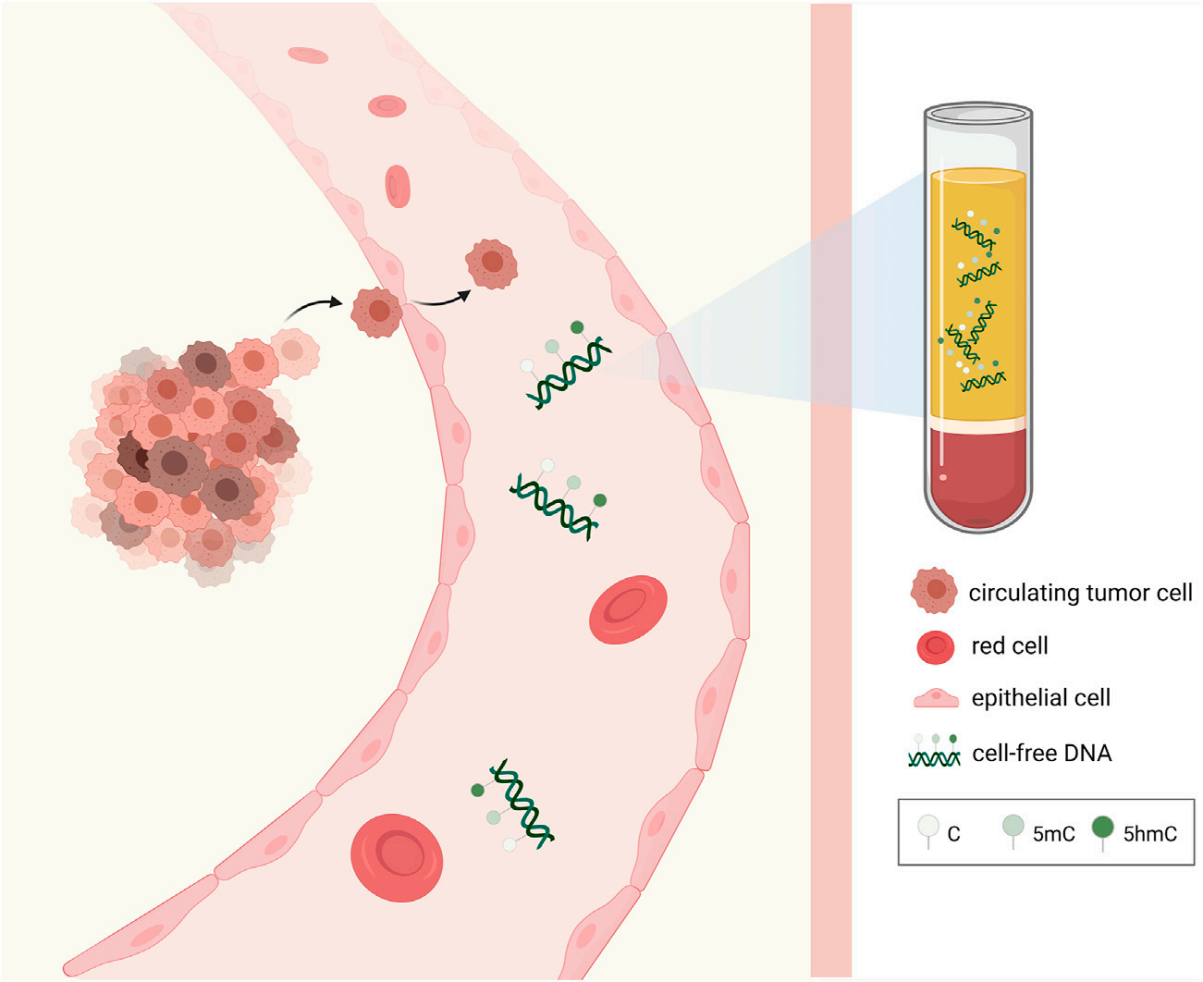
Epigenetic alterations of cell-free DNA extracted from the blood. Tumor-derived epigenetic alterations including 5mC, 5hmC of cfDNA obtained from the blood via liquid biopsy can be utilized as novel biomarkers for cancer diagnosis, prognosis, and treatment.

Liquid biopsy, along with the detection of circulating tumor DNA (ctDNA) has been revolutionizing the cancer screen and treatment (Tuaeva et al., 2019). ctDNA is tumor-derived fraction of circulating cell-free DNA (cfDNA) that can reflect both genetic and epigenetic profiles of cancer (Wan et al., 2017) (Figure 2). The ctDNA-based liquid biopsy offers substantial advantages over traditional biopsies (Chen and Zhao, 2019; De Rubis et al., 2019; Shohdy and West, 2020). First, it is minimally invasive and it avoids the risks of tissue biopsy such as tumor seeding (Diaz and Bardelli, 2014). Second, it could provide dynamic surveillance of genetic or epigenetic changes during cancer development (Diaz and Bardelli, 2014; Wan et al., 2017). Third, it could help capture the heterogeneity of the tumor, as ctDNA fragments are derived from all clones of the cancer (Diaz and Bardelli, 2014). The detection of 5hmC in cfDNA has been reported and it is increasingly fueled by the development of high-throughput sequencing methodology (Li et al., 2017). For instance, considerable researchers used 5hmC-Seal method to capture 5hmC patterns in colorectal, gastric, hepatocellular and lung cancers (Li et al., 2017; Zhang et al., 2018; Cai et al., 2019). However, 5hmC as a biomarker in liquid biopsy of cancer remains unclear, which warrants more exploration.

In this review, we focus on the value of 5hmC detection in cfDNA as an emerging biomarker in cancer management and possible mechanisms. Specifically, we discuss how 5hmC exerts its role in the early detection, prognosis, and treatment of various malignancies. We further overview the mechanisms underlying aberrant 5hmC patterns, TETs, and carcinogenesis. Notably, we emphasize that cfDNA-based liquid biopsy combined with genome-wide sequencing of 5hmC patterns may serve as an efficient tool to implement 5hmC into clinical applications in the era of precision medicine.

## Deoxyribonucleic Acid 5-Hydroxymethylation Promotes Early Detection of Cancer

Early detection of cancer leads to better outcome (Wan et al., 2017; Shohdy and West, 2020). The detection of epigenetic alterations including 5hmC could promote early detection of cancer (Jankowska et al., 2015). 5hmC modification is tissue-specific and disease-specific (Laird et al., 2013; Sproul and Meehan, 2013; Jankowska et al., 2015), and has been reported in the process of multiple precancerous pathogenesis. For instance, the increase and decrease of 5hmC level were respectively associated with the cervical precancerous lesions (Su et al., 2019) and the breast precancerous lesions (Zhang et al., 2020). 5hmC was also involved in hepatocellular carcinoma (HCC), melanoma and esophageal cancer (Lian et al., 2012; Tian et al., 2018; Liu et al., 2019a; Krais et al., 2019). Furthermore, as cfDNA can be identified in a minimal invasively and dynamically way in body fluids, recent studies demonstrated that 5hmC in cfDNA could act as a novel biomarker in cancer detection (Li et al., 2017; Song et al., 2017), taking a substantial step toward precision medicine.

5hmC in cfDNA has been detected in multiple malignancies, including HCC (Cai et al., 2019), colorectal cancer (CRC) (Gao et al., 2019), lung cancer (Zhang et al., 2018), gastric cancer (Li et al., 2017) and pancreatic ductal adenocarcinoma (PDAC) (Song et al., 2017; Guler et al., 2020). A reduced global 5hmC level in HCC was found to be positively correlated with tumor stage (Chen et al., 2013; Chen et al., 2017; Liu et al., 2019a) and it could be observed even in chronic hepatitis without significant methylation (Wang et al., 2019). Cai et al. reported the promising value of 5hmC in cfDNA in early detection of HCC (Cai et al., 2019). The research analyzed the genome-wide 5hmC patterns in cfDNA among 2,554 Chinese HCC patients, showing that the 5hmC profile in cfDNA could effectively distinguish early HCC patients from healthy controls and liver cirrhosis. Moreover, the research designed a weighted model comprised of a 32-gene-based 5hmC marker panel, which is proved to outperform alpha-fetoprotein (AFP) in early detection of HCC (Cai et al., 2019). Likewise, lung cancer also exhibits a global loss of 5hmC (Jin et al., 2011). Song et al. found that the global level of 5hmC in cfDNA progressively decreased from early-stage lung cancer to metastatic lung cancer, indicating that 5hmC in cfDNA could contribute to the early detection and surveillance of lung cancer (Song et al., 2017). Another study based on a Chinese cohort reported that 5hmC of specific genes in cfDNA have a higher sensitivity than traditional biomarkers, including CEA, CA125, NSE, and CYFRA21-1 in the early detection of non-small cell lung cancer (NSCLC) (Zhang et al., 2018). However, 5hmC pattern in cfDNA from this study was found to exhibit global gains in gene bodies as well as promoter regions in NSCLC patients (Zhang et al., 2018). Guler et al. revealed that 5hmC peaks of cfDNA in promoter region in PDAC patients were lower than those in non-cancer patients, whereas it was significantly higher over 3′UTR, transcription termination sites (TTS) and intron regions in PDAC patients, respectively. 5hmC profile in PDAC tissues was reported could classify PDAC cfDNA, which indicates the cfDNA could mirror the 5hmC enrichment observed in tumor (Guler et al., 2020). Another study reported that 5hmC peaks gained in cfDNA were present in the gene body of representative genes in colorectal cancer tissues, compared with patients of precancerous adenomas and healthy donors, such as *BCL11A*, *KCNK9* and *NTSR1* (Gao et al., 2019). Similarly, Xiao et al. determined the genome-wide distribution of 5hmC in cfDNA, indicating that the CRC patients have the widest distribution of 5hmC, whereas the healthy donors own the narrowest distribution, and the precancerous adenomas have the moderate distribution (Xiao et al., 2021). 5hmC in cfDNA was also reported associating with molecular subtypes in non-Hodgkin lymphoma (Chiu et al., 2021). Therefore, accumulating evidences indicate that 5hmC in cfDNA, both the distribution and global level, could be served as robust biomarkers in early detection of cancer, though further research is still warranted.

Despite the fact that cfDNA could be identified in the blood, only a small fraction is from the tumor and it remains difficult to identify aberrant epigenetic profiles precisely (Jankowska et al., 2015; Shen et al., 2018). Specifically, though 5hmC profiles are apparent in tumors at an early stage, it is still challenging to detect them by next-generation sequencing, as ctDNA itself is at an imperceptible level, especially in early stage cancer (Locke et al., 2019; Kilgour et al., 2020). It is reported that ctDNA in asymptomatic cancer is not detectable based on current methodology using typical 10 ml of blood, hindering its application in cancer early detection (Fiala and Diamandis, 2018). However, the emerging 5hmC-Seal technology has been utilized to capture genome-wide profiling of 5hmC and it proved to be a highly sensitive and robust approach for genome-wide profiling of 5hmC that requires only very limited input of DNA (< 10 ng) (Han et al., 2016; Applebaum et al., 2020; Guler et al., 2020). Also, other innovative methodologies permit the implementation of ctDNA in early-stage cancer detection (Liu et al., 2019b; Hu et al., 2019). Therefore, although 5hmC in ctDNA is difficult to detect, rapidly evolving methodology permit its application in early detection of cancer.

## Deoxyribonucleic Acid 5-Hydroxymethylation Predicts Cancer Prognosis

In the era of precision medicine, increasing studies demonstrated the potential of 5hmC, TET protein family as potential clinical biomarkers to predict the prognosis of cancer, however, the evidence remains inadequate.

The decrease of 5hmC level is closely correlated with poor prognosis in multiple malignancies, including HCC (Liu et al., 2013; Liu et al., 2014; Liu et al., 2019a), CRC (Zhong et al., 2018), NSCLC (Liao et al., 2016), breast cancer (Tsai et al., 2015), gastric cancer (Yang et al., 2013; Frycz et al., 2014), papillary thyroid carcinoma (PTC) (Tong et al., 2019), glioblastomas (GBM) (Orr et al., 2012), esophageal carcinoma (EC) (Shi et al., 2016), cholangiocarcinoma (CCA) (Dong et al., 2015; Bai et al., 2021). In HCC, it has been reported that loss of 5hmC was positively correlated with tumor size, AFP level as well as poor overall survival (Liu et al., 2013). In patients with breast cancer, especially the ER/PR-negative subtype, the reduction of 5hmC was found strongly associated with poor disease-specific survival (DSS) and disease-free survival (DFS) (Tsai et al., 2015). In patients with PTC, one study revealed that the level of 5hmC was lower in those with lymph node metastasis than those without, which indicated that 5hmC may be a useful tool in predicting the lymph node involvement of PTC (Tong et al., 2019). Moreover, the reduction of 5hmC level showed its prognostic value in WHO grade II diffuse astrocytomas (DA) and WHO grade IV GBM (Kraus et al., 2015; Zhang et al., 2016a; Johnson et al., 2016). Low 5hmC was correlated with increased mortality and poor survival in patients with GBM (Orr et al., 2012), and it could act as a biomarker in predicting tumor infiltration instead of ki67 and H3S10p (Kraus et al., 2015). It has been revealed that the loss of 5hmC may affect normal gene transcription, which could in turn promote abnormal proliferation of tumor cells, and eventually affect the survival in cervical squamous cell carcinoma (CSCC) (Zhang et al., 2016b). On the other hand, high level of 5hmC was associated with less invasive tumor phenotype and better prognosis in colon cancer (Zhong et al., 2018), and it was also displayed as a predictor of post-operative recurrence in *TMPRSS2-ERG* negative prostate cancer (Strand et al., 2015). Therefore, the detection of 5hmC levels in various cancers serves as a promising approach in predicting cancer prognosis.

The detection of 5hmC signature in cfDNA may provide a novel tool in predicting cancer outcomes. For instance, Song et al. observed a gradual decrease of 5hmC in cfDNA in early stage and advanced metastatic lung cancer, suggesting that such stage-dependent 5hmC loss in cfDNA may be a powerful tool for outcome prediction (Song et al., 2017). Cai et al. reported a novel HCC score predicting the prognosis of HCC, which consisted of cfDNA, 5hmC, and protein biomarkers [des-gamma-carboxy prothrombin (DCP) and AFP]. The results indicated that a higher HCC score was positively associated with the risk of recurrence and metastasis (Cai et al., 2021). In addition to the global level of 5hmC, the distribution of 5hmC in cfDNA also process a prognostic value. For instance, Chiu et al. reported that in diffuse large B-cell lymphoma (DLBCL), the distinct 5hmC profile in cfDNA could reflect origin of cell, thereby distinguishing different subtypes of DLBCL, and it was associated with the events of recurrence, retreatment, or death in DLBCL (Chiu et al., 2019; Chiu et al., 2021). Collectively, these studies indicate that 5hmC has a great potential as a biomarker of cancer progression, while the detection of 5hmC in cfDNA could contribute to the development of liquid biopsy in cancer.

In summary, DNA 5-hydroxymethylation in the genome is a novel prognostic biomarker in multiple cancers. The detection of 5hmC in cfDNA could help predict the outcomes of patients with cancer. However, the research on the detection of 5hmC still needs to be explored unremittingly by researchers in the future.

## Targeting Deoxyribonucleic Acid 5-Hydroxymethylation in Cancer

Targeting DNA demethylation has great potential in cancer treatment. Lian et al. suggested that feasible manipulation of overexpressing TET2 could restore the 5hmC level and further suppress tumor growth and invasion in melanoma, unlatching a novel therapeutic avenue for melanoma by targeting 5hmC generating pathway to reestablish 5hmC levels or landscape in melanoma cells (Lian et al., 2012). Another study showed that TET2 plays a key role in controlling the numbers and survival of slow-cycling cancer cells (SCCCs) through controlling the expression of cell death-related genes that regulate TNF-α signaling and restraining its proapoptotic signaling (Puig et al., 2018). Slow-cycling is a temporary state preserving potential cancer initiation and increased chemo-resistance. High TET2 activity and 5hmC level were associated with resistance to chemotherapy, thereby increasing the risk of recurrence of patients. Thus, 5hmC could be served as a biomarker for identifying the increased chemo-resistant SCCCs and TET2 could be potentially targeted for treatment of SCCCs (Puig et al., 2018). Interestingly, high expression of TET1 leads to global hydroxymethylation in T-cell acute lymphoblastic leukemia (T-ALL), which protects cells from DNA damage and thereby promoting leukemic growth. Bamezai et al. outlined that PARP could induce TET1 expression, thus TET1 could be pharmacologically targeted via application of PARP inhibitor Olaparib, which showed great therapeutic potential in T-ALL treatment (Bamezai et al., 2021). Fisetin, a natural flavonol, was reported to inhibit the activity of TET1, reduce 5hmC levels in CCNY/CDK16 promoter, and further restrain the invasion and progression of renal cancer stem cells. It indicated that TET1 could be targeted and 5hmC could be a potential indicator for the treatment of fisetin in renal cancer (Si et al., 2019). Besides, Vitamin C, a cofactor for TETs to enhance 5hmC conversion, is noticeably involved in the process of DNA demethylation (Dickson et al., 2013; Minor et al., 2013; Sant et al., 2018). Sant et al. reported that the reduced expression of Na^+^-dependent Vitamin C transporter 2 (SVCT2) was a primary cause of 5hmC loss in breast cancer (Sant et al., 2018). Treatment of vitamin C might make up for the deficiency of SVCT2, thus restoring 5hmC levels via activating TET activity, and inducing apoptosis in breast cancer cells (Sant et al., 2018). Moreover, it has been suggested that 5hmC or 5hmC/5mC ratio might be a potential biomarker of differentiation and proliferation in pituitary neuroendocrine tumor, which could also be used as a biomarker of the effect of decitabine (Szabó et al., 2020). Therefore, we propose that 5hmC in cancer treatment could be a promising target, although more research is warranted.

5hmC could be a biomarker in cancer immunotherapy, especially in predicting immune cell exhaustion and immune evasion. Johnson et al. found a significant age-related differentially hydroxymethylated region occurred in the intronic region of *TOX2,* which is associated with CD8^+^ T cell exhaustion. It belongs to a family of transcription factors that modify chromatin structure during T cell development (Johnson et al., 2020). Researchers reported that circTRIM33-12 could inhibit immune evasion of HCC by sponging oncogenic miR-191 and upregulating TET1 expression, which lead to a significant increase of 5hmC levels (Wang et al., 2019). Similarly, Taylor et al. found that the immune evasion subtype of lung squamous cell carcinoma has significant enhanced EGFR signaling, which further boosted the expression of the transcription factor ETS1. ETS1 could upregulate miR-29b, which result in the downregulation of TET1 and subsequent reduction of 5hmC level (Taylor et al., 2016). In another study, TET2 was reported promoting tumor progression in melanoma via *ARG1*, an immunosuppressive gene in tumor-associated macrophages. *ARG1* embraced a low level of 5hmC in Tet2-null bone marrow derived macrophages (BMDM) (Pan et al., 2017). Targeting TET2 and its interactors (e.g., Fe^2+^, 2-OG, Vitamin C) could regulate the activity of B cells, T cells, and macrophages, which could contribute to immunotherapy of cancer (Jiang, 2020). Luchtel et al. reported that ascorbic acid treatment could increase the level of 5hmC in CD8^+^ T cells and reinforce their cytotoxicity against lymphoma cells (Luchtel et al., 2020). Therefore, the 5-hydroxymethylation of DNA could be a promising biomarker and a target of cancer immunotherapy, although further studies are required.

Targeting different oncogenes in the pathways of DNA demethylation, including TETs and its cofactors, may be potential strategies in cancer treatment in the future. 5hmC in these processes can be taken as a valuable biomarker for the response to multiple therapies. In terms of immunotherapy of cancer, 5hmC provides a new approach for immunotherapy-sensitive tumors, especially in overcoming immune cell exhaustion and cancer immune evasion. Although TET or 5hmC plays a critical role in cancer treatment, the involvement of TET and 5hmC in inhibiting cancer development is still far from clarified. Some attempts have been undertaken to develop pharmaceutical drugs targeting TET or its correlated signaling pathways in cancer development. For instance, TET1 has been considered as a critical oncogenic epigenetic regulator in acute myeloid leukemia (AML), which plays its role by promoting the expression of oncogenic targets, including *HOXA9*, *MEIS1* and *PBX3*, and suppressing the expression of tumor suppressor miR-22 (Huang et al., 2013; Huang et al., 2016; Jiang et al., 2016). Jiang et al. found that application of selective inhibitors (NSC-370284 and UC-514321) could directly target STAT/TET1 signaling, which was an effective and promising therapeutic strategy to treat TET1-high AML, especially when combined with standard chemotherapy (Jiang et al., 2017). Therefore, targeting TET1 signaling might be a considerable strategy in AML treatment. Moreover, TET1 and 5hmC were reported associating with an obesity-linked pathway driving cancer stem cells in triple-negative breast cancer, which providing new understanding on the cancer prevention and treatment (Bao et al., 2020). With the development of liquid biopsy, the detection of cfDNA provides new pathways for cancer treatment (Goodall et al., 2017; Siravegna et al., 2017). For instance, a recent study investigated the alteration of 5hmC profiles during the treatment of neuroblastoma. The study showed that 5hmC profiles in cfDNA changed over time, paralleled with changes in metastatic disease burden as well as patients’ response to therapy (Applebaum et al., 2020). Thus, the detection of 5hmC profiles in cfDNA may be a promising biomarker that can be widely applied in monitoring the response to cancer treatment in future.

## Mechanisms Underlying Deoxyribonucleic Acid 5-Hydroxymethylation in Cancer

Although the alteration of DNA 5-hydroxymethylation can be utilized as potential biomarkers in cancer screen, prognosis, and treatment, the mechanisms underlying these epigenetic alterations remain obscure. Exploring how the alteration of TETs and 5hmC play roles in tumorigenesis and tumor progression could accelerate the development of cancer therapy.

**FIGURE 3:**
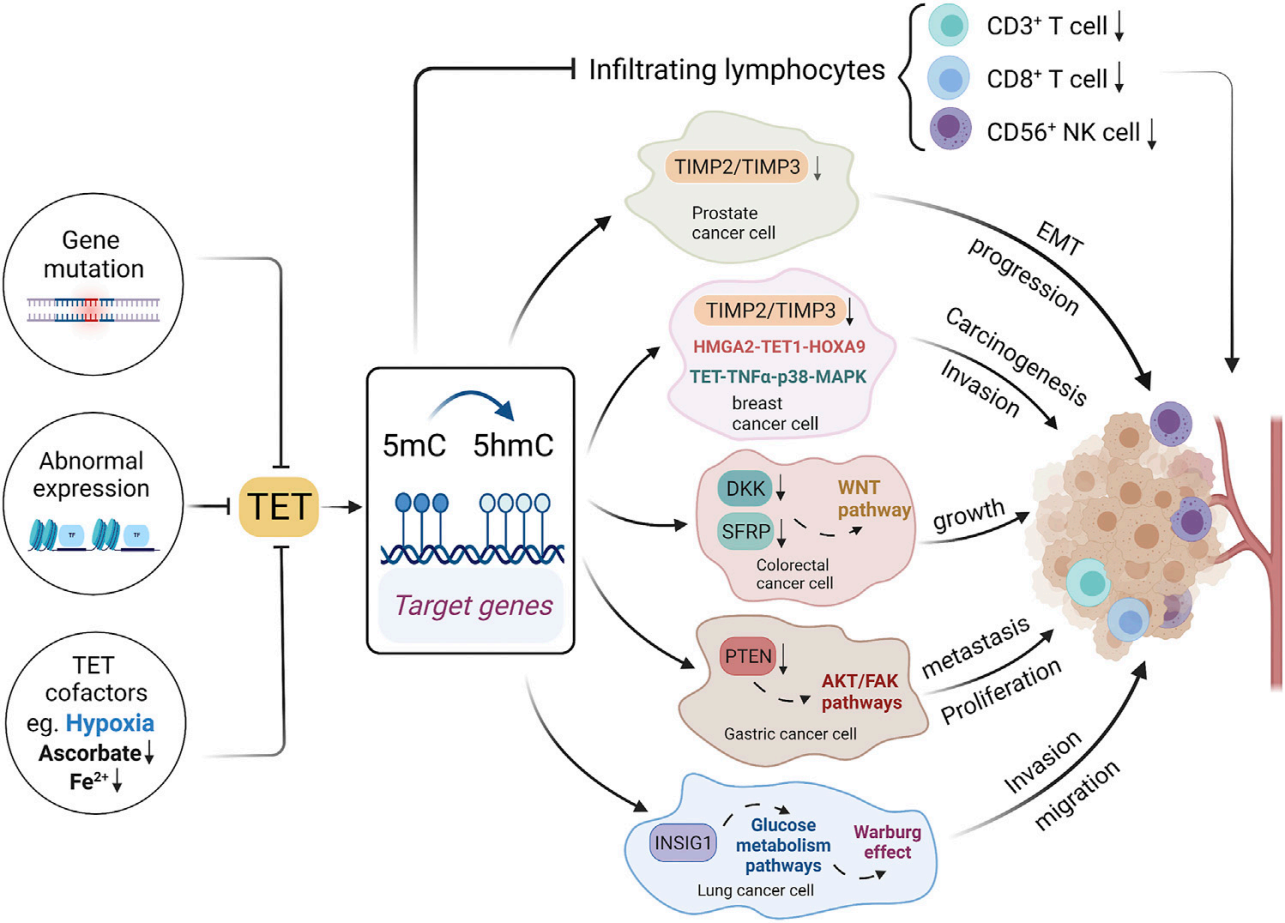
Mechanisms underlying 5hmC and TET alterations in cancer. Gene mutations, abnormal expression and cofactors could affect TET expressions, thereby affecting DNA hydroxymethylation. The TET gene family could affect the tumor infiltrating lymphocyte, and it could also affect tumor development through multiple signal pathways.

The abnormal expression of TETs, or inhibition of TETs cofactors lead to the alteration in the level and localization of 5hmC, impacting the cancer development and anti-tumor immunity (Koivunen and Laukka, 2018) (Figure 3). Compared with TET2 which is frequently mutated in hematopoietic malignancies (Huang and Rao, 2014; Jeschke et al., 2016), TET1 mutation is a rare occurrence in cancer (Thomson and Meehan, 2017). Instead it is found both methylated and transcriptionally suppressed *in vitro* in multiple malignancies, though whether it is the cause of hypermethylation is still unclear (Wu and Brenner, 2014). In addition to the genetic or transcriptomic disruption, the activity of TETs can be regulated by its substrates, cofactors and post-translational modifications (Thomson and Meehan, 2017). It has been found that patients with hematological malignancies and gliomas often harbor mutations in *IDH1* an *IDH2*, which can convert 2-OG into 2-hydroxyglutaraye (2-HG) and thereby inhibit the catalytic activity of TET (Figueroa et al., 2010; Xu et al., 2011). The loss of 5hmC in cancer can be further explained by the post-transcriptional regulation of TET. For instance, the IDAX protein, an interacting partner of TET2 that was often proved to be mutated in tumors, leads to TET2 degradation via caspase activation (Ko et al., 2013). However, decrease in hydroxymethylation in promoters of tumor suppressor genes is scarcely reported during carcinogenesis, which may require further investigations.

TET1 is usually known as a tumor suppressor, which could inhibit cancer progression via various pathways (Hsu et al., 2012; Fu et al., 2014; Neri et al., 2015; Yan et al., 2020). TET1 directly binds to the promoter of the target genes, catalyzing 5mC to 5hmC and ultimately activating gene transcription (Pei et al., 2016). In accordance with this mechanism, one study showed that the downregulation of TET1 could reduce the hydroxymethylation at the promoter of tumor suppressor gene *AJAP1*, subsequently activating WNT/β-catenin pathway and promoting the development of bladder cancer (Yan et al., 2020). TET1 loss in prostate cancer and breast cancer was found contributing to the hypermethylation of tissue inhibitors of metalloproteinases genes (*TIMP2* and *TIMP3*), which resulted in the epithelial-mesenchymal transition (EMT) and tumor progression (Hsu et al., 2012). Another study reported the HMGA2-TET1-HOXA9 axis could regulate breast cancer cell invasion, and it holds a prognostic signature in predicting patient survival (Sun et al., 2013). TET1 is negatively regulated by HMGA2, a chromatin remodeling factor that is highly expressed in most epithelial cancers. TET1 positively modulated HOXA9 by directly binding to or inducing the histone binding of H3K4Me3 to the HOXA gene promoter regions, leading to local demethylation and gene transcription in breast cancer. Additionally, the downregulation of TET1 was reported to occur at the early stage and its expression could inhibit the proliferation of cancer cells in CRC (Neri et al., 2015). TET1-mediated demethylation induces a 5hmC increase at the promoter regions of *DKK* and *SFRP* genes, which were upstream inhibitors of WNT signaling pathway, hence TET downregulation can lead to cancer growth via repressing these inhibitors of WNT pathway (Neri et al., 2015). Similar mechanism was also reported in pancreatic cancer (Wu et al., 2019). Likewise, TET1 inhibits proliferation and metastasis of gastric cancer cells through demethylating PTEN promoter regions, which subsequently suppress the PTEN-inhibited AKT and FAK pathways (Pei et al., 2016). Besides DNA 5-hydroxymethylation mediated by TETs in tumor-suppressor genes, several oncogenes which encompass an acquisition of 5hmC were also reported. One study demonstrates that the 5hmC landscape in pancreatic ductal adenocarcinoma (PDAC) peaks in the gene that encodes the bromodomain-containing transcription factor BRD4, overlapping with active enhancer marks such as H3K4me1, and 5hmC acquisition by BRD4 enhancer is found to be a potential mechanism for regulating oncogenic activation (Bhattacharyya et al., 2017). In one word, 5hmC under the regulation of TETs is involved in the initiation and progression of tumors via various pathways, which is of great significance for its status as a promising biomarker in the field of precise oncology.

Besides, it is exciting to find that TETs and 5hmC changes are involved in immune signaling pathways, which may boost antitumor immunity. One study reported that in basal-like breast cancer (BLBC), the activation of nuclear factor кB (NF-кB) could suppress TET1 by binding to its promoter, highlighting a novel epigenetic-immunity connection (Collignon et al., 2018). NF-кB is a transcription factor involved in the activation of immune cells and regulation of immune signaling pathways (Gerondakis and Siebenlist, 2010; Hoesel and Schmid, 2013). Compared to TET1-high patients, low TET1 expression and subsequent 5hmC loss were associated with high infiltration of immune cells and better survival in BLBC (Collignon et al., 2018). Thus, the level of 5hmC could act as a marker of the immune response targeting tumor cells. TET2 also plays a substantial role in antitumor immunity. One study demonstrated that TET2 depletion could reduce Th1-type chemokines and PD-L1 expression by mediating the IFN-γ/JAK/STAT pathway, thereby decreasing tumor-infiltrating lymphocytes such as CD3^+^ and CD8^+^ T cell, and eventually it could compromise the efficacy of anti-PD-1/PD-L1 immunotherapy (Xu et al., 2019). Moreover, the 5hmC level in this study was positively correlated with the expression of chemokines and PD-L1 in tumor cells, infiltration of antitumor immune cells including CD3^+^ T cells, CD8^+^ T cells, and CD56^+^ NK cells, and the response towards anti-PD-1/PD-L1 therapy. Thus, TETs and 5hmC could potentially be predictors of antitumor immunity, which may help boost the immunotherapy of cancer.

Oxygen is an essential element of TETs catalysis during demethylation (Schito and Semenza, 2016). Several studies have reported the association between TETs activity and hypoxia in different cancer (Mariani et al., 2014; Tsai et al., 2014; Wu et al., 2015). One study reported that hypoxia could reduce 5hmC levels in tumor-suppressor gene promoters by directly impairing TET oxidative activity in oncogenic process (Thienpont et al., 2016). However, in another study, hypoxia could conversely increase the expression of TET1, TET3 as well as 5hmC level in promoters, leading to carcinogenesis and poor prognosis of breast cancer patients via a TET-TNFα-p38-MAPK signaling (Wu et al., 2015). Interestingly, it was also reported that hypoxia-induced TET1 was associated with lipid metabolism in inducing EMT and cancer metastasis. ‘Hypoxia-induced TET1 could increase the 5hmC level in the promoter of insulin induced gene 1 (*INSIG1*), the main regulator of cholesterol biosynthesis (Tsai et al., 2014). The knockdown of *INSIG1* could abolish the activation of mesenchymal genes, mitigating the activity of migration and invasion of lung cancer cells, and it could even decrease gene expression in the glucose metabolism pathway that contribute to the hypoxia-induced Warburg effect in tumor cells (Tsai et al., 2014). Thus, TET1 may play a crucial role in regulating cholesterol and glucose metabolism in tumor cells, although the mechanisms need to be further explored. In summary, the relationship between aberrant 5hmC patterns and carcinogenesis relies on both transcriptional state of TETs and the hypoxic environment in cancer cells (Thomson and Meehan, 2017). With further exploration of the mechanisms underlying 5hmC and carcinogenesis, the identification of new therapeutic targets and medications could be a promising field in the precise medicine of cancer.

## Conclusion and Perspective

In this review, we overviewed the current research on use of DNA hydroxymethylation as a novel biomarker in the era of precision medicine. 5hmC has been highly prevailing in recent years in cancer diagnosis and prognosis. Different from genetic mutations encoded in DNA sequence, DNA hydroxymethylation is potentially reversible, which holds great promise in cancer treatment by using pharmaceutical intervention to recover normal epigenetic patterns (Thomson and Meehan, 2017). It is worth considering to effectively translate cancer-associated 5hmC patterns into clinical practice in the field of oncology.

With the development of genome-wide sequencing assays in the last few years, 5hmC profiling can be detected via ctDNA or cfDNA-based liquid biopsies, which are effective, minimally invasive tools for cancer early detection and progression surveillance. Although several studies demonstrated the detection methods of 5hmC signatures in cfDNA remain crude (Zeng et al., 2019). Another challenge is that circulating DNA from scarce tumor sub-populations can only be shown at an extremely low level in cfDNA, restraining its detection (Chen et al., 2013). This should be considered when designing novel assays and evaluating the role of 5hmC patterns in cancer by liquid biopsy. Overall, 5hmC could be a robust and pivotal biomarker in the era of liquid biopsy and precision medicine.

## Abbreviations

5mC, 5-methylcytosine; TSGs, Tumor suppressor genes; 5hmC, 5-hydroxymethylcytosine; TET, ten-eleven translocase; 5fC, 5-formylcytosine; 5caC, 5-carboxylcytosine; 2-OG, 2-oxoglutarate; ctDNA, circulating tumor DNA; cfDNA, cell-free DNA; HCC, hepatocellular carcinoma; CRC, colorectal cancer; AFP, alpha-fetoprotein; NSCLC, non-small cell lung cancer; PDAC, pancreatic ductal adenocarcinoma; TTS, transcription termination sites; PTC, papillary thyroid carcinoma; GBM, glioblastomas; EC, esophageal carcinoma; CCA, cholangiocarcinoma; DSS, disease-specific survival; DFS, disease-free survival; DA, diffuse astrocytomas; CSCC, cervical squamous cell carcinoma; DCP, des-gamma-carboxy prothrombin; DLBCL, diffuse large B-cell lymphoma; SCCCs, slow-cycling cancer cells; AML, acute myeloid leukemia; SVCT2 Na+-dependent Vitamin C transporter 2; BMDM, bone marrow derived macrophages; TIMP, tissue inhibitors of metalloproteinases; EMT, epithelial-mesenchymal transition; PDAC, pancreatic ductal adenocarcinoma; BLBC, basal-like breast cancer; NF-&kcy;B, nuclear factor &kcy;B; TDG, Thymine DNA glycosylase; BER, base-excision repair; 5hmU, 5-hydroxymethyluracil.
